# Cross-Domain Federated Data Modeling on Non-IID Data

**DOI:** 10.1155/2022/9739874

**Published:** 2022-09-09

**Authors:** Baobao Chai, Kun Liu, Ruiping Yang

**Affiliations:** College of Computer Science and Engineering, Shandong University of Science and Technology, Qingdao 266590, China

## Abstract

Federated learning has received sustained attention in recent years for its distributed training model that fully satisfies the need for privacy concerns. However, under the nonindependent identical distribution, the data heterogeneity of different parties with different data patterns significantly degrades the prediction performance of the federated model. Additionally, the federated model adopts simple averaging in the model aggregation phase, which ignores the contributions of different parties and further limits the model performance. To conquer the above challenges, we propose a new cross-domain federated data modeling (CDFDM) scheme by combining the attention mechanism. Firstly, to mitigate the poor model performance caused by data heterogeneity, we propose a shared model that adjusts the number of shared data assigned to users according to their data size, which effectively alleviates data heterogeneity while avoiding shared data from overwriting the user's individual data features. Then, we introduce the attention mechanism in the model aggregation phase, which assigns weights to users according to their contributions, thus improving the model performance. Finally, we conducted a series of experiments on two real-world datasets (MNIST and CIFAR-10). The results show that our CDFDM outperforms existing schemes in both nonindependent identical distribution conditions. Furthermore, in terms of model prediction accuracy variation during the training phase, our approach is more stable.

## 1. Introduction

In recent years, machine learning [1] has achieved notable results in various fields, such as recommendation [2] and traffic prediction [3]. The emergence and continual advancement of neural networks, in particular, have led to more indepth research in machine learning. Machine learning's tremendous performance is strongly reliant on massive amounts of training data. Nonetheless, these training data are typically dispersed across multiple parties that are isolated from one another and pushed to form data silos. For rising privacy concerns, it is challenging to collect and organize these data including private information [4]. Federated learning (FL) [5] allows data silos to be broken down by training cooperatively while ensuring that data are stored locally for all parties.

FL [6] has garnered considerable attention since its inception for its distributed training property. However, there are still issues with data heterogeneity in the federated setting. Persons of different ages, for example, will generate diverse daily data, and even among people of the same age, factors such as area and occupation might have an impact on data distribution, resulting in data heterogeneity between users. Unfortunately, in that circumstance, the federated model's performance suffers dramatically [7]. As a consequence, we require a methodology to address the federated model's performance degradation caused by data heterogeneity.

Many existing works have investigated the challenge of nonindependent identical (Non-IID) distribution of data under federated learning [8]. Many algorithms take Non-IID into account, as well as changes in communication capability, computational power, etc. [9, 10]. Simultaneously, due to the significant heterogeneity of data among users and the inconsistency of user criteria for model performance, it is impossible for a single global model to suit the needs of all participants. Consequently, personalized federated learning [11] is another approach to dealing with Non-IID. Using shared model parameters as the initial parameters of the model to replace the process of random initialization of the model parameters results in improved prediction at the start of the model and speeds up model convergence. However, in [12], the authors only use the shared model parameters as initial parameters, with no further use of the shared model parameters in the subsequent training process. Therefore, the shared model in this manner is not beneficial for the model's parameter learning.

Although the aforementioned approaches improve the federated learning model from various angles, there are still certain issues that must be addressed. Not only is there data heterogeneity among various parties but the amount of data preserved by each party varies due to storage and computational restrictions. Once the parties' data patterns and amounts change, so does their significance in the global model. This is easy to comprehend since local models with large data volumes will have a greater influence on the global model. Nevertheless, when aggregating models to obtain the global model, most methods use averaging, which overlooks differences in user contributions and results in a limited model performance increase.

To address the aforementioned issues, we propose a novel cross-domain federated data modeling scheme (CDFDM) to address the challenges of data isolation and heterogeneity across various parties. The main contributions are summarized as follows:

- We propose a shared model and utilize it as the initial model parameters to substitute the random initialization process, which speeds up model convergence and alleviates data heterogeneity. In addition, we integrate the shared model into the global model to fully exploit the value of shared data and increase the global model's accuracy.
- In the model aggregation stage, we leverage the attention mechanism to quantify the differences between the local model and the global model and give weights to them based on the quantified results. This weight represents the weight of the local model in the global model after aggregation, and this method accounts for disparities in the contributions of distinct objects and effectively improves the global model's performance.
- We conduct a series of experiments and comparative analysis to investigate the effects of different parameters on model performance under common nonindependent identically distributed partitioning patterns, and the experimental and analytical results demonstrate the efficacy of our proposed scheme.

The rest of the paper is organized as follows: Section 2 presents the research related to our work.  Section 3 describes our proposed federated model in detail followed by the introduction of the experimental setup and a comparative analysis of the experimental results in Section 4. Finally, a summary is given in Section 5.

## 2. Related Work

The widely known aggregation approach in FL, FedAvg [13], often fails when data are heterogeneous over a local client. Xu et al. [14] proposed a modified federated averaging (FedAvg) algorithm later, which was also unable to address the issue of data heterogeneity. Zhao et al. [12] discovered that the accuracy reduction can be explained by the weight divergence and can be quantified by the Earth mover's distance (EMD). To tackle the statistical heterogeneity, they proposed a heuristic approach to improve training accuracy on Non-IID data by sharing a global subset with all the devices.

Based on the abovementioned study, Wang et al. [15] considered that different parties may conduct different numbers of local steps each and proposed a normalized averaging method, which eliminates objective inconsistency while preserving fast error convergence to ensure that the global updates are not biased. Li et al. [10] improved the local objective, which directly limits the size of local updates. Specifically, it introduces an additional regularization term in the local objective function to limit the gap between the local model and the global model. Karimireddy et al. [16] proposed a new algorithm which introduces variance among the parties and applies the variance reduction technique in its local updates to account for “client-drift.”

In contrast to the previous studies, Shin et al. [17] worked at the data layer by directly augmenting raw Non-IID data while obscuring the features of the original data through encoding to improve model performance. Li et al. [18] proposed FedBN to conquer feature shift before model aggregation, in which the client batch-norm layers are updated locally without communicating to the server. In addition, the authors demonstrated that FedBN converges faster than the classical Fedavg scheme. In [19], the authors proposed FedAMP, a new method employing federated attentive message passing to facilitate similar clients to collaborate more. The FedAMP not only has stronger convergence characteristics but also uses a deep neural network as a personalized model for the client, which further improves the model performance even more.

In [20], a novel federated learning framework is proposed for learning a shared data representation across clients and unique local heads for each client to tackle Non-IID, which can effectively minimize the problem dimension per client. In [21], a novel weight similarity-based client clustering (WSCC) method is proposed, in which clients are grouped into different groups based on their dataset distribution to tackle the nonindependent and identical distribution. It leverages the cosine distance of the client's weight parameters to estimate dynamic clustering iteratively and automatically without the requirement for auxiliary models or further data transfer.

Although the approaches discussed above have approached the problem of nonindependent identical distribution in federated learning from various angles, they all use averaging in the aggregation stage, neglecting guest differences and restricting model performance.

## 3. Proposed Model

In this section, we first introduce our proposed shared model and then elaborate on our proposed federated model.

### 3.1. Shared Model

**Figure 1:**
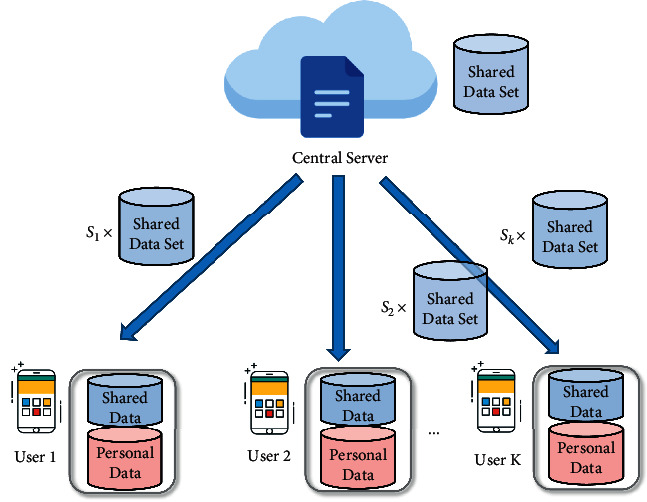
The shared model.

In order to alleviate the Non-IID problem, the method of the shared subset is proposed in [12]. Firstly, a dataset is selected by a central server, and then the same proportion of data from the selected dataset is allocated to all users participating in the training. Users mix the obtained data with their own data for training, which helps to ease the problem of data heterogeneity to some extent. However, in the case of data heterogeneity, different users have different amounts of data but the amount of shared data allocated to all users is equal, and the number of local iterations and global communication of users is the same in the experimental setup, which inevitably leads to a certain degree of the diminished role of the local model of users with small original data in the global model. To overcome the aforementioned issue, we ameliorate the method in [12] and propose a new shared model with the following model architecture as shown in Figure 1.

As illustrated in Figure 1, the central server first selects a batch of data as a shared dataset and then randomly selects a portion of data from the dataset to distribute to users. In order to ensure the balance between shared data and users' private data, the amount of data allocated to different users varies and *S*_*i*_ denotes the proportion of data received by users. Users with fewer data will receive fewer shared data, and our ultimate focus is to minimize the influence of shared data on the user's own data features. We assume that the ratio of shared data received by users to their own original training data is 0.3.

After the shared data are distributed, the system obtains the initial parameters *ω*_*t*_ of the model through the initialization operation and distributes them to each user, where *ω*_*t*_ denotes the global model parameters for the *t* th round of communication. After receiving *ω*_*t*_, users train and update *ω*_*t*_ with their own data to obtain a local model *ℓ*_*k*_^*t*+1^ for the user *κ*. Following local training, all users upload their local model to the central server, which performs model aggregation. Because the users' data are already fused with shared data, the degree of heterogeneity is relatively reduced, so the aggregation rule adopts the classical federated average algorithm (FedAvg [13]) as follows:

(1) $$\begin{array}{c} M_{s}^{t+1}\leftarrow \frac{1}{K}\overset{K}{\underset{\kappa =1}{\sum }}l_{\kappa }^{t+1}\text{,} \end{array}$$

where *M*_*s*_^*t*+1^ denotes the shared model parameters obtained after *t*+1 rounds of communication.

### 3.2. Federated Model

**Figure 2:**
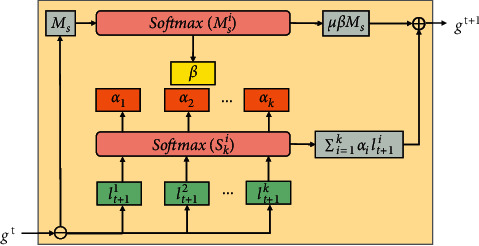
The federated model.

Model aggregation is the most significant part of federated learning; to measure the contribution of users in the global model, we used the attention aggregation approach [22]. The hierarchical attention federated aggregation scheme is depicted in Figure 2 , which displays only one iteration of the process before obtaining the global model. The purpose of our iteration and model aggregation was to find a global model with good generalization performance for all users.

The abovementioned problem can be regarded as a parametric solution problem, and in order to make full use of the shared model, in addition to replacing the random initialization process with the shared model, the shared model is also incorporated into the global model, so our objective optimization function is redefined as follows:

(2) $$\begin{array}{c} \underset{g_{t+1}}{\text{argmin}}\overset{K}{\underset{\kappa =1}{\sum }}\left(\frac{1}{2}\alpha_{\kappa }L{\left(g_{t},l_{t+1}^{\kappa }\right)}^{2}+\frac{1}{2}\mu \beta \left(g_{t},M_{s}\right)\right)\text{.} \end{array}$$

In equation (2), *g*_*t*_ and *ℓ*_*t*+1_^*k*^ denote the global model parameters at the *t* th iteration and the local model parameters of the user *κ* at the *t*+1 th iteration, respectively. *L*(·) function is used to find the difference between the two models. *α*_*κ*_ represents the attentive weight of the global model for user *κ*. *β* represents the attention weight of the shared model *M*_*s*_, and *μ* is used to manually adjust the proportion of the shared model in the global model. It is vital to note that both *α*_*κ*_ and *β* are not fixed values but will be trimmed throughout the iterative process until the model converges or the iteration ends.

We assume that the model parameters have *l* layers, and we utilize the Euclidean distance between the global model and the local model to express the difference between them, as shown in equation (3). *g*^*i*^ denotes the parameter at the layer *i* of the global model. Similarly, *ℓ*_*i*_^*k*^ indicates the parameter value of the local model of user *κ* at layer *i*.

(3) $$\begin{array}{c} {\left[s_{\kappa }^{i}\right]}_{i=1}^{1}=\left\|g^{i}-l_{\kappa }^{i}\right\|\text{.} \end{array}$$

After obtaining the difference between the local model and the global model through equation (3), we then utilize the softmax to calculate the attention weight of the user *κ* at a layer *i*. We repeat the abovementioned procedure to obtain the weight value of the user *κ* at each layer and finally obtain *α*_*κ*_, as shown in equation (4). Similar to the preceding step, in order to make full use of the shared model, we also obtain the weight value *β* for the shared model.

(4) $$\begin{array}{c} \alpha_{k}^{i}=\frac{e^{s_{\kappa }^{i}}}{\sum_{k=1}^{k}e^{s_{\kappa }^{i}}}\text{.} \end{array}$$

After obtaining *α*_*κ*_ and *β*, we derive the corresponding gradient from equation (2), as follows :

(5) $$\begin{array}{c} \nabla =\overset{\kappa }{\underset{\kappa =1}{\sum }}\alpha_{\kappa }\left(g_{t}-l_{t+1}^{\kappa }\right)+\mu \beta \left(g_{t}-M_{s}\right)\text{.} \end{array}$$

For all the *K* users who participated in the training, the algorithm optimization process is shown in the following equation, where *λ* denotes the step size. The global model *g*_*t*+1_ is finally obtained after the *t*+1 iteration.

(6) $$\begin{array}{c} g_{t+1}\leftarrow g_{t}-\lambda \left(\overset{k}{\underset{k=1}{\sum }}\alpha_{k}\left(g_{t}-l_{t+1}^{k}\right)+\mu \beta \left(g_{t}-M_{s}\right)\right)\text{.} \end{array}$$

## 4. Evaluation of Experiments

In this section, we describe the datasets and parameters used in the experiments, followed by comparison and analysis of the experimental outcomes.

### 4.1. Datasets

In this work, we test the performance of our proposed model using two publicly available datasets, MNIST [23] and CIFAR-10 [24]. Both MNIST and CIFAR-10 datasets contain ten different types of images and are mostly used for image classification tasks. The MNIST dataset contains 70000 gray pictures, 60000 of which are used as the training set to train the neural network model and the remaining 10000 as the test set to test the model's performance. The CIFAR-10 dataset contains 60000 colored images separated into ten different categories. 50000 photos are used to train the network model in the experiment, while the remaining 10000 images are utilized to test the model's performance.

In order to model the heterogeneous data distribution, the training data are divided among users according to their classes throughout the training process, and there are two primary types of data division as follows: (1) 1-class Non-IID categorizes the complete dataset by category, and each user receives data from C classes, where C ∈{1,10} and (2) 2-class Non-IID categorizes and sorts the images according to the category they belong to, then each user is randomly assigned *y* images, where the value of *y* ranges from 120 to 600, and the remaining images are finally distributed to the user with the least number of images. The above two data partitioning models not only simulate the heterogeneous situation of user data patterns but also take into account the difference in user data volume, which is more realistic. Additionally, in the 2-class Non-IID, we divide the user data into two parts, one from the sorted dataset and the other taken at random from the entire disordered dataset. The percentage of data drawn from the sorted dataset is controlled by P. Assuming that the number of photos allocated to a user in the Non-IID (2) is 350 and P is equal to 0.8 at this moment, only 280 images are drawn from the sorted dataset, while the remaining 70 images are drawn from the full disordered dataset.

### 4.2. Experimental Setting

In this work, we train the dataset with a convolutional neural network to train the dataset whose architecture is consistent with that in [13]. We use the following notations in our experiments for our algorithm: we utilize SGD as the model optimizer, and B represents the batch size in SGD for each round of training. *E* is the number of rounds of local iterations executed by federated learning users. For example, if *E* equals 10, all users involved in training will first execute 10 rounds of local training before passing the model parameters to the central server. The following parameters are used for our experiments: for MNIST dataset, *B* = 10 and 100, *E* = 1 and 5, learn rate *η*  = 0.01, and decay rate = 0.995; for CIFAR-10, *B* = 10 and 100, *E* = 1 and 5, learn rate *η* = 0.1, and decay rate = 0.992. Besides, *μ* is used to control the scale of the shared model, which can be adjusted according to the actual situation. We set the total number of users participating in training *N* to 100 and then randomly select users with proportion *f* from 100 users for training and model update in each round, with default *f* equal to 0.1, that is, 10 users are randomly selected from 100 users for local training and used for global model update in each round.

The meanings and settings of some basic parameters in the experiment are shown above. The batch size and the number of local iterations both have a significant impact on the model performance. There are also some more parameters that need to be mentioned. In the first data division method, each user can get C classes of images, where C can be any integer between 1 and 10. In order to observe the effect of C on the model performance, we conduct the following experiments for the parameter C.

### 4.3. Results and Analysis

**Figure 3:**
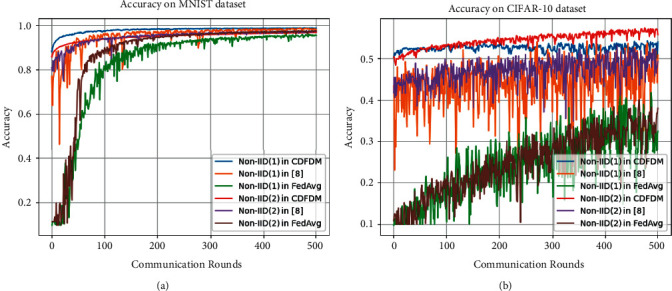
Accuracy on two datasets. We set C equal to 1 under Non-IID (1) and P to 0.8 in Non-IID (2). (a) MNIST and (b) CIFAR-10.

To demonstrate the effectiveness of our proposed scheme, we conducted a series of experiments and thoroughly studied the experimental results. First, we conducted experiments in the two previously indicated data partition types, and the results are given in Figure 3. Above all, we can observe that the initial model performance of our scheme is superior to the other two schemes. The greater initial performance of our scheme is mostly due to our shared model, which allocates shared data to users according to their data volume, highlighting the local model more than the undifferentiated allocation method in [12] and thus yielding better results. In contrast, the FedAvg scheme uses random initialization to obtain the initial model, resulting in the worst starting performance. Moreover, Figure 3 shows that our scheme is more stable than the other two schemes, notably in Figure 3(b), where the volatility of the other two schemes is more visible.

**Figure 4:**
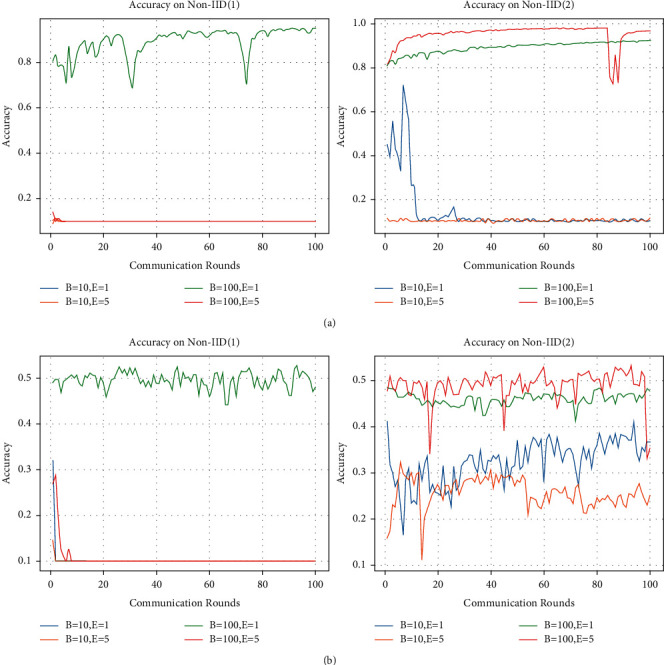
Accuracy on two datasets with different batch size (B) and local iteration (*E*). We set C equal to 1 under Non-IID (1) and P to 0.8 in Non-IID (2). (a) MNIST and (b) CIFAR-10.

In order to verify the effect of batch size B and the number of local iterations *E* on the model performance, we conducted experiments with different parameters of the two cases, and the experimental results are presented in Figure 4. It should be noted that, except for modifying the values of B and *E*, all other parameters remain unchanged as can be seen from the tests shown in Figure 3.

Figure 4(a) depicts the prediction accuracy of our CDFDM for *B* and *E* on the MNIST dataset using two different data partitioning patterns. We set C to 1 in Non-IID (1), indicating that the training data for each participating user contains only one class of images. The left panel in Figure 4(a) shows that the model is valid only when *B* = 100 and *E* = 1. After 100 rounds of training, the model can achieve a prediction accuracy of 94%, while the model fails under all other three combinations of parameter values. Specifically, due to the high degree of heterogeneity in the data, if B is set too low, the model cannot get enough information on the data distribution at each training; hence, the model will not work when B is set to 10, regardless of whether *E* is set to 1 or 5. Increasing the number of local iterations in federated learning is intended to improve the user's local model's representation of its data distribution features. When the data heterogeneity is too large, increasing the number of local iteration rounds causes the global model derived after aggregation to no longer fit the local data distribution. Hence, the model will still fail when *E* is equal to 5, even if *B* is set to 100.

We set P to 0.8 in Non-IID (2), which means that 20 % of the training data for each participating user is selected at random from the MNIST dataset. When *B* is set to 10, a problem similar to the Non-IID (1) setting develops, which is likewise caused by the small value of B. After 100 rounds of training, with *B* set to 100 and *E* to 1, the model prediction accuracy can reach 92%. The difference is that when B is set to 100 and *E* to 5, the model prediction accuracy rises to 96%.  Figure 4(b) depicts our CDFDM's model prediction accuracy on the CIFAR-10 dataset under two data partitioning patterns with regard to *B* and *E*. Except for the differing datasets, all parameters are consistent with Figure 4(a). When *B* is set to 100 and *E* to 1, the model prediction accuracy in Non-IID (1) can reach 47% after 100 rounds of training, but the model fails in the other three parameters. The right panel of Figure 4(b) shows that when *B* is set to 100, the prediction accuracy is much higher when *E* is set to 5 than when *E* is set to 1. Yet regardless of whether *E* is 1 or 5, the model prediction accuracy is lower when *B* is set to 10.

**Figure 5:**
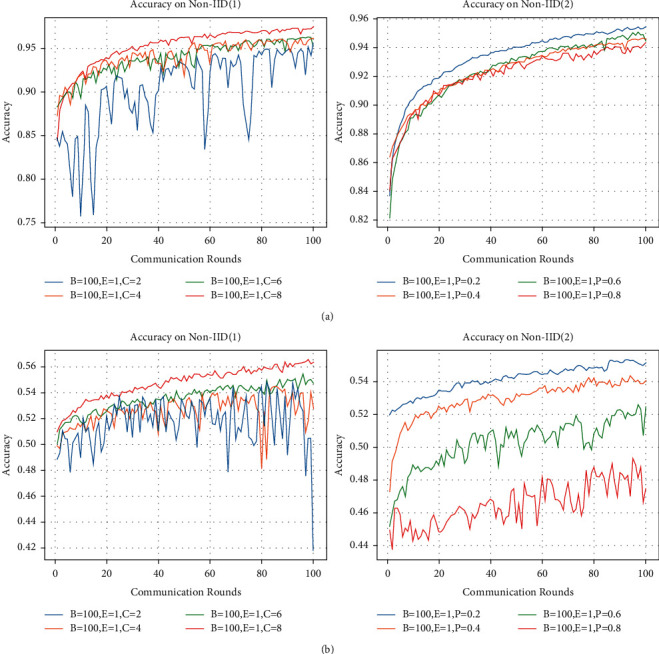
Accuracy on two datasets. We fix the batch size B and the local iterations (E) and change the values of C and P in Non-IID (1) and Non-IID (2). (a) MNIST and (b) CIFAR-10.

According to the results in Figure 4, we find that the trend of our CDFDM's prediction accuracy is similar for both datasets and both data partitioning patterns. Furthermore, the influence of the identical *B* and *E* settings on model performance varies when the degree of heterogeneity of the user data varies in both data partitioning patterns. To further validate the relationship between CDFDM and *B* and *E*, we performed experiments on model prediction accuracy where the user training data were heterogeneous to varying degrees under the two data partitioning patterns, and the experimental results are presented in Figure 5.

First, we conducted experiments for each of the two data partitioning patterns on the MNIST dataset, and the results are presented in Figure 5(a). We set the values of *B* and *E* to 100 and 1, respectively, to study the influence of data heterogeneity on prediction accuracy. When *C* is set to 8, the model prediction accuracy approaches 97% under Non-IID (1), implying that the user training data used in training contains 8 different classes of images. And, it is clear that, as C increases, the model prediction accuracy improves.

A larger value of P in Non-IID (2) represents a smaller number of randomly selected data from the user training data, indicating a higher degree of data heterogeneity. The model's prediction accuracy is the highest at *P* and is set to 0.2, 95% because the data heterogeneity is the lowest at this point. Following that, we conducted experiments on the CIFAR-10 dataset, and the results are presented in Figure 5(b). Under Non-IID (1), after 100 rounds of training, it can be seen that the model's prediction accuracy is highest when *C* is 8, and this accuracy drops as the value of *C* lowers, and the model's prediction accuracy is poorest when *C* is 2, and then it shows a continuous downward trend. In Non-IID (2), the model prediction accuracy is highest when *P* is set to 0.2 and declines as *P* grows. As the *P* value rises, so does the heterogeneity of the user training data, resulting in worse prediction accuracy. It should be mentioned that while the picture structure of the CIFAR-10 dataset is more complex than that of the MNIST dataset, the prediction accuracy of the CIFAR-10 dataset is lower. However, as demonstrated in Figure 5, the less the heterogeneity of the user training data, the greater the model's prediction accuracy under the same *B* and *E* settings.

**Figure 6:**
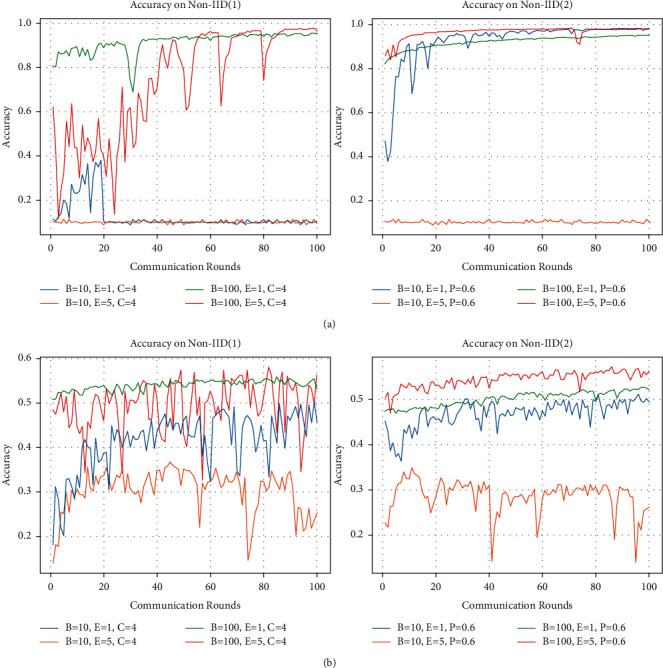
Accuracy on two datasets. We fix the values of *C* and *P* in Non-IID (1) and Non-IID (2); we change the batch size B and the local iterations E. (a) MNIST and (b) CIFAR-10.

Combining the previous two scenarios, we conclude that when the data is much more diverse, increasing the number of local iterations or decreasing the number of samples would produce poorer results. We followed up with a two-step experiment to corroborate our findings. First, we used the prior step to lower the degree of heterogeneity in the data and set it up for both data partitioning scenarios.We increased the total number of categories C assigned to the training data by the user in the first partitioning type, and we increased the proportion of shared data assigned to the user in the second partitioning type. Then, we choose one of the two data partitioning cases with relatively moderate data heterogeneity to conduct the experiments, and the results are shown in Figure 6. For the first type of data partitioning, we take *C* = 4, and the results on both datasets show that increasing the number of local iterations does not help the model performance when the size of samples is small. However, when the data heterogeneity is minimal and the size of samples is big, increasing the number of local iterations on the model yields marginal gains. For the second data partitioning type, we set P to be 0.6. When the size of samples is small, increasing the number of local iterations leads to a decrease in the model performance. When the number of samples is big, the model's improvement via local iterations is also small.

In summary, our proposed federated model achieves not only improved prediction accuracy but also a more stable convergence process for both types of data partitioning. Besides, we demonstrated how the degree of data heterogeneity affected model performance by adjusting different parameters, and we explained why our strategy performs better.

## 5. Conclusion

In this paper, we proposed an attention-based strategy for the federated data modeling scheme CDFDM to address the problem of low model accuracy in federated learning due to data heterogeneity. Our scheme included a shared model, which alleviated the problem of data heterogeneity by distributing shared data. Simultaneously, in the model aggregation phase of federated learning, we developed an attention mechanism that can quantify the weight of different users' local models in the global model and increase the global model's prediction accuracy. Finally, we conducted a series of experiments on two real-world datasets, and the results demonstrated that our scheme outperformed the other two methods in terms of prediction accuracy. Furthermore, the experimental results indicated that our scheme provided better stable model prediction performance during the training process.

The model prediction accuracy of our CDFDM changes very gradually during the training process, and the experimental results also support this view. However, there is one problem in practical application and that our model does not outperform the traditional method in terms of prediction accuracy. To overcome the aforementioned issue, we will investigate an upgraded federal learning model in future for the nonindependent homogeneous distribution problem in order to increase the model prediction accuracy.

## Data Availability

The data used to support the findings of this study are included within this article.
